# Toward Coalescing Gene Expression and Function with QTLs of Water-Deficit Stress in Cotton

**DOI:** 10.1155/2015/892716

**Published:** 2015-06-18

**Authors:** Hirut Kebede, Paxton Payton, Hanh Thi My Pham, Randy D. Allen, Robert J. Wright

**Affiliations:** ^1^USDA-ARS Crop Genetics Research Unit, Stoneville, MS 38776, USA; ^2^USDA-ARS Cropping Systems Research Laboratory, Lubbock, TX 79415, USA; ^3^Department of Plant and Soil Science, Texas Tech University, Lubbock, TX 79409, USA; ^4^Department of Biochemistry and Molecular Biology, Oklahoma State University, Stillwater, OK 73401, USA

## Abstract

Cotton exhibits moderately high vegetative tolerance to water-deficit stress but lint production is restricted by the available rainfed and irrigation capacity. We have described the impact of water-deficit stress on the genetic and metabolic control of fiber quality and production. Here we examine the association of tentative consensus sequences (TCs) derived from various cotton tissues under irrigated and water-limited conditions with stress-responsive QTLs. Three thousand sixteen mapped sequence-tagged-sites were used as anchored targets to examine sequence homology with 15,784 TCs to test the *hypothesis* that putative stress-responsive genes will map within QTLs associated with stress-related phenotypic variation more frequently than with other genomic regions not associated with these QTLs. Approximately 1,906 of 15,784 TCs were mapped to the consensus map. About 35% of the annotated TCs that mapped within QTL regions were genes involved in an abiotic stress response. By comparison, only 14.5% of the annotated TCs mapped outside these QTLs were classified as abiotic stress genes. A simple binomial probability calculation of this degree of bias being observed if QTL and non-QTL regions are equally likely to contain stress genes was *P*_(*x* ≥ 85)_ = 7.99  × 10^−15^. These results suggest that the QTL regions have a higher propensity to contain stress genes.

## 1. Introduction

Although cotton (*Gossypium* spp.) exhibits moderately high tolerance during vegetative development, water-deficit stress is one of the major limiting factors in its production. Advancements in genome mapping and functional genomics provide a powerful resource for the genetic dissection of abiotic stress tolerance in crop plants [1]. Large-scale genome projects have generated a mass of knowledge regarding the genome organization and function of stress-responsive genes in plants [2]. Searchable databases and analytic tools available to the research community offer the capacity to query these data. These comparative tools from related fields enable the identification of genes and gene products and may reveal functional relationships between a genotype and observed phenotype [3, 4]. Hence, there is an opportunity to make direct and meaningful comparisons from data generated by quantitative trait loci (QTLs) mapping and genome-wide expression analysis to provide solutions for crop improvement.

Several studies have identified QTLs responsible for drought stress-related traits in cotton. Saranga et al. [5, 6] described a substantial number of QTLs that explained phenotypic variation in physiological variables such as osmotic potential, carbon isotope ratio, canopy temperature, and chlorophyll a and chlorophyll b content, and measures of crop productivity like dry matter, seed cotton, harvest index, boll weight, and boll number, under water-limited and/or well-watered conditions. Water-deficit stress during cotton boll and fiber development affects fiber quality characteristics. QTLs for fiber length, length uniformity, elongation, strength, fineness, and color detected under water-limited and well-watered conditions have also been reported [7]. QTL mapping alone does not provide knowledge regarding the mechanisms and pathways involved in water-deficit stress tolerance, or about the multitude of genes involved in a plant's response to water-deficit stress [8]. Linking information from QTL mapping and genome-wide expression experiments offers a powerful approach to identify and characterize the key pathways and the genetics underlying water-deficit stress tolerance [4, 8]. Integration of QTL information, physiological knowledge, and gene expression data is a significant step towards understanding genes controlling physiological responses that affect production and quality under stressful conditions.

A community-wide effort produced 185,198 Expressed Sequence Tags (ESTs) from 30 cDNA libraries, sampling a variety of tissues and developmental stages, including several subjected to abiotic stresses such as chilling temperatures and water-deficit treatment (http://www.agcol.arizona.edu/cgi-bin/pave/Cotton/index.cgi/) [9]. Subsequently, this EST collection and others have provided a wealth of sequence data for microarray-based investigation into a number of key biological processes in cotton including fiber development, pathogen response, and water-deficit stress response [10–14]. Even though these arrays do not provide complete transcriptome coverage, particularly for stress-related genes, together with the assembled EST database, described by Udall et al. [9], they have provided a foundation for more robust functional analysis of a multitude of developmental responses in cotton. Additionally, the ability to integrate expressed sequence data with QTLs data may allow the identification of functional regions on chromosomes that contribute to variability in quantitative traits.

A long-term goalis to explore the regulatory networks that control the expression of stress responsive genes. The principal aim of this study was to identify cotton genes implicated in water-deficit stress by integrating information generated by QTL mapping and genome-wide expression analysis. This research examined the utility of genome sequence information as a means to link functional gene expression and QTL knowledge. To bridge this information 3016 mapped sequence-tagged sites (STS) were used as anchored targets to examine sequence homology with 25,118 cotton ESTs derived from various tissues under irrigated and water-limited conditions. Our* hypothesis* is that putative stress-responsive genes will colocalize to QTLs associated with phenotypic variation for stress-related traits more frequently than with other genomic regions not associated with these QTLs. Forty-four genes that appear to be functional orthologs of genes associated with stress tolerance responses, differentially expressed in response to water-deficit treatment, and mapped within a QTL likelihood interval were identified as candidate genes. This approach provides a strategy for combinatorial genomic analyses to identify candidate genes that will be useful tool for genetic optimization of fiber productivity and quality under water-limited conditions.

## 2. Materials and Methods

### 2.1. EST Assembly and Annotation

Tentative consensus sequences (TCs) were generated from 25,118 cotton ESTs derived from 10 libraries: boll (irrigated and water-limited), stem, seedling (irrigated, water-limited, and cold-stress), ovary, and etiolated cotyledon tissue (http://www.agcol.arizona.edu/cgi-bin/pave/Cotton/index.cgi/) (Table 1). Assembly was conducted using SeqMan, Lasergene Version 5.07 (DNASTAR, Inc., Madison, WI). A threshold of 90% identity with 80 bp minimum overlap was used in the assembly. The BLASTX (Basic Local Alignment Search Tool, X) function was used for sequence annotation against publicly available databases of the National Center for Biotechnology Information (NCBI). The default matrix BLOSUM 62 and a cut-off of 1 × 10^−6^ were used in the BLASTX search.

### 2.2. In Silico EST Mapping

A genetic map representing the hypothetical ancestral diploid genome (Consensus Map), constructed from the homoeologous chromosomes of tetraploid (A_*t*_ and D_*t*_) and diploid (D) cotton [16, 17], was used to map the ESTs. Chromosomes 1 to 13, indicated in this study, are the consensus chromosomes on the Consensus Map. Sequence homology between the mapped sequence tagged sites (STS) and the TCs was used as a basis to determine the putative genetic location of TCs on the Consensus Map. Each TC was aligned to 3016 genetically mapped STS [16, 17]. These STS loci were derived from cDNAs (abscission tissue and drought-stressed tissue from* G. hirsutum*, 7–10-day fiber from* G. arboreum*, putative gene function, disease resistance gene analogs, and* Arabidopsis* ESTs) and genomic DNA. The BLASTN function was used to search sequences homologous to STSs with known chromosomal locations using a threshold of 90% similarity and a minimum of 100 bp overlap.

Our previous research [13] identified 2106 stress-responsive transcripts (ESTs), 879 classified as stress-induced, 1163 stress-repressed, and 64 showing reciprocal expression patterns in leaf and root exposed to water-deficit stress. These transcripts were identified from the Cotton Oligonucleotide Microarray (v1) (http://www.cottonevolution.info/), composed of 12,006 microarrays derived from an assembly of more than 180,000* Gossypium* ESTs sequenced from 30 unrelated libraries. The map position of these additional 2106 stress-responsive transcripts was also investigated as described previously.

### 2.3. Quantitative Trait Locus (QTL) Alignment

The relationship among ESTs and QTLs was investigated based on knowledge from a meta-analysis of polyploid cotton QTLs [15]. Using conserved markers to align the different genetic maps, a total of 432 QTLs were integrated into the Consensus Map. Among them 39 QTLs, 18 related to plant physiology (osmotic potential (OP), carbon isotope ratio (*δ* ^13^C), chlorophyll *a* and chlorophyll *b* content (*Chl-a *and* Chl-b*) and canopy temperature (CT)), 17 to plant productivity (dry matter (DM), harvest index (HI), seed cotton (SC), and boll weight (BW)), and 4 to fiber quality (fiber strength (FS), fineness (FF), and length (FL)) were detected only under water-limited conditions [5–7] (Table 2). The seventeen genomic regions that contain these QTLs were the focal point for comparison with the gene expression and gene ontology data that follows. Any TC or EST that mapped within the 99% (2-LOD) confidence interval of a QTL was deemed to colocalization within the QTL.

### 2.4. Cotton-*Arabidopsis* Synteny

Comparative analysis linked to synteny-based and expression-based information may provide clues about specific genes and families involved in QTL networks that respond to abiotic stress. Comparative analysis was conducted on significant QTL regions to deduce the cotton-*Arabidopsis* synteny relationship and examine the correspondence between the 39 QTLs and* Arabidopsis* abiotic stress responsive genes. Each QTL region was aligned with the corresponding cotton consensus map (http://www.plantgenome.uga.edu/cotton/StartFrame.htm) based on conserved marker loci [15]. Markers that flanked the 99% confidence interval (2-LOD) of each QTL were used in this alignment. Consensus fragments were subjected to both FISH [18] and CrimeStatII [19] analysis to identify putative regions of synteny with* Arabidopsis* [16]. Gene ontologies were determined for the* Arabidopsis* genes showing correspondence with the cotton QTL regions.

## 3. Results

### 3.1. EST Mapping

Comparisons of expression data with mapped QTLs were carried out using a subset of stress responsive ESTs. This was done by integrating ESTs with gene expression and QTL data through a Consensus Map [17]. Initially, 25,118 ESTs from several diverse libraries (Table 1) were assembled into 15,784 TCs that represented 13,097 singletons and 2687 assemblies. Interestingly, only 539 unique cotton transcripts were found within these 10 stress-treated cDNA libraries.

The sequence of each TC was compared to the composite set of 3016 STSs on the Consensus Map [17]. The putative map location of 1,906 TCs was determined based on this homology. In all, 815 loci contain a significant level of homology to assign the putative map position of at least one TC. Because unique TCs showed homology to several regions of the same gene it was expected that mapped loci would contain multiple TCs and indeed this was observed at 462 (57%) loci.

### 3.2. Association of TCs and Differentially Expressed Genes with QTLs

The correspondence of mapped TCs and QTLs revealed that 349 of the 1906 mapped TCs colocalized within the 99% confidence interval of at least a single QTL (Table 2). Putative functions (BLASTX) could be assigned to 243 of which 85 (35.0%) were annotated as genes involved in plant responses to abiotic stress (Table 3). By comparison, only 14.5% (160 out of 1,104) of the annotated ESTs mapped outside the QTL interval were classified as abiotic stress genes (Table 3). A simple binomial probability calculation of this degree of bias being observed if QTL and non-QTL regions are equally likely to contain stress genes—“85 or more stress genes from 243 annotated genes,” where *p* = 0.145—yields a likelihood of only 7.99 × 10^−15^, suggesting that the QTL regions have a higher propensity to contain stress genes. The enrichment of stress-related ESTs that map to stress-related QTLs could not be explained by chance; thus this observation supports the hypothesis that stress responsive genes map to stress-associated QTLs at higher frequency than to non-QTL regions. The 85 TCs mapped to 33 STS loci and 29 stress-related QTLs (Table 4(a)).

We have previously examined the drought stress transcriptome in cotton exposed to field capacity and water-limited conditions [13]. Transcript profiling experiments in leaf and root tissues revealed 2106 stress-responsive transcripts, 879 classified as stress-induced, 1163 stress-repressed, and 64 showing reciprocal expression patterns. In this study, 158 genes (84 stress-induced and 74 stress-repressed) were mapped, of which 34 (14 induced, 17 repressed, and 3 reciprocal expression) colocalized within the 99% confidence interval of at least a single QTL (Tables 4(a) and 4(b)). Thirteen (13) showed homology with at least one TC annotated as a plant stress genes and mapped within a QTL region. This number is less than expected and likely due to the limitation in the number of stress-specific genes represented on the microarray.

### 3.3. Candidate Gene Selection

**Figure 1:**
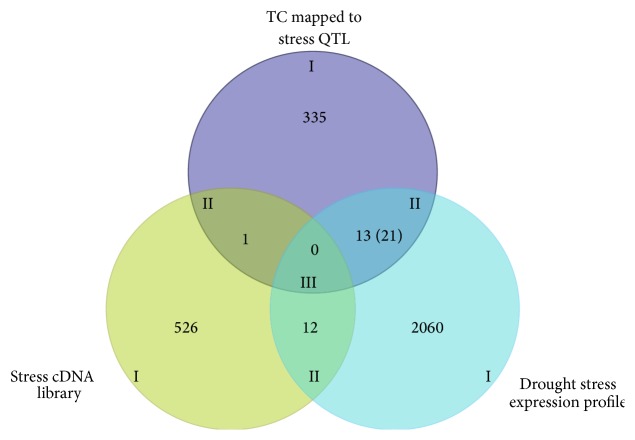
A Venn diagram showing the levels of candidate gene identification. Three categories including unique transcripts in stress-treated cDNA libraries, differential expression profile, and TCs mapped to a stress QTL defined level I candidate genes. Genes associated with multiple Level I categories (overlapping regions) define Level II and Level III candidates. The number in parentheses indicates unique differentially expressed drought stress ESTs that mapped to stress QTLs.

Candidate genes were identified based on the merger of mapping data, putative function, and expression (microarray or RT-PCR). Three levels of candidate genes have been considered in this study. A schematic presentation of the candidate gene selection/classification process is depicted by a Venn diagram (Figure 1). Three categories including unique transcripts in stress-treated cDNA libraries (539), differential expression (2106), and colocalization with a QTL (349) defined “Level I” candidate genes (Figure 1). The overlap of two or more Level I categories define “Level II” and “Level III” candidates genes. Levels I and II candidates with putative stress-related gene ontology contain a prime (′) designation (Table 4). From these categories of possible candidate genes those which show homology to known stress-related genes, colocalized within stress-related QTLs, and/or were differentially expressed in response to drought stress were further selected. Based on this criterion, 44 genes were identified as possible candidates that may have influence on the associated QTLs (Tables 4(a) and 4(b)).

### 3.4. Cotton-*Arabidopsis* Synteny

An appreciable degree of synteny and colinearity between cotton and* Arabidopsis* provides a means to employ genomics approaches to look for additional clues as to the identities of genes influencing the cotton plant's response to abiotic stress. Twenty-six cotton QTLs on Chrs. 2, 4, 5, 7, 8, 10, 12 and 13 were associated with 51 stress-related* Arabidopsis* genes (Table 5). Forty-eight (48) stress related* Arabidopsis *genes that could be putatively mapped within a QTL region. Four of these* Arabidopsis *genes were homologous to and fell on the same map location with four of the cotton candidate genes. These include genes that respond to drought, salt and cold stress, and abscisic acid stimulus and that function in the regulation of transcription.

### 3.5. Relationship among Functional and Structural Data

Osmotic potential is an important indicator of plant water status. Two QTLs on Chrs. 1 and 11 influenced OP (Table 2). Four additional OP QTLs on Chrs. 4, 7, 10, and 13 mapped to regions that contained other physiological (*Chl-a* and *δ* ^13^C) and productivity (SC, HI, and BW) QTLs. These QTLs contain 136 TCs of which 35 have putative gene function and 9 were differently expressed (Table 2). Thirty-six TCs representing 13 loci were classified as Level I′, II, or II′ candidate genes (Table 4(a)). Carbon isotope ratio (*δ* ^13^C) has been used to assess differential responses to water-deficit stress. A total of 5 QTLs influenced *δ* ^13^C (Table 2), of those, four (Chrs. 2, 3, 7, and 8) were associated with QTLs for physiological (*Chl-a, Chl-b*, and OP), productivity (HI), and fiber (FF) traits. A total of 114 TCs delineate these QTL regions. Twenty-one TCs representing 7 loci were classified as Level I′ or II′ candidate genes (Table 4(a)).

Plant productivity traits mapped to Chrs. 5, 6, 7, 10, 12, and 13. A total of 185 TCs mapped to these QTL regions. In many cases multiple productivity QTLs fell within the same genomic region. Three separate regions (Chrs. 7, 10, and 13) contain corresponding QTLs for SC, HI, and OP. Twenty-one loci, represented by 58 TCs, associated with these QTLs were classified as Level I′, II, or II′ candidates (Table 4(a)). Fiber quality QTLs on Chrs. 2, 7, and 12 contained 10 TCs classified as Level I′, II, or II′ candidate genes (Table 4(a)).

## 4. Discussion

We have mapped 1906 cotton TCs on a Consensus Map that represents the hypothetical ancestor diploid genome [16]. Forty-four candidate genes implicated in water-deficit stress response were identified by merging structural and functional data. The association of these candidate genes with QTLs that influence physiology, plant productivity, and fiber quality traits in cotton under drought stress conditions was investigated. We have used cotton-*Arabidopsis* comparative analysis to examine association of stress-related genes in* Arabidopsis *with the drought stress cotton QTLs. The Consensus Map depicts the inferred marker arrangement along the genome of the common ancestor that gave rise to the diploid progenitors of tetraploid cotton about 5–7 million years ago [16, 20]. This resource sets the stage for exploring syntenic relationships and thus fosters study of correspondence between the cotton QTLs and genes from* Arabidopsis*. Indeed,* Gossypium* and* Arabidopsis* are thought to have shared common ancestry about 83–86 million years ago [21], and cotton may be the best crop outside of the Brassicales in which to employ “translational genomics” from* Arabidopsis*.

The mapping of TCs revealed that 815 STS loci contain a significant level of homology to assign the putative map position of at least one TC. Because unique TCs showed homology to several regions of the same gene it was expected that mapped loci would contain multiple TCs and indeed this was observed at 462 (57%) loci. Gene duplication would compound this effect, considering that homoeologous loci in tetraploid cotton would map to a single locus on the Consensus Map, which was inferred to resemble the DNA marker arrangement of the hypothetical ancestor of the two subgenomes of tetraploid cotton [16]. Interestingly, 59% (1128) of the TCs mapped to multiple loci on the Consensus Map. This observation may support a growing body of evidence suggesting that an ancient gene duplication event (polyploidy) has shaped the genome organization of what is considered diploid cotton [15, 16]. If this hypothesis is correct, then one would expect an elevated level of gene redundancy in the Consensus Map. However, we cannot exclude the possibility that multigene families may account for the association of the TCs to multiple loci.

The scientific merit of this research serves as a framework in which information can be combined to simplify a more complex problem. Absolutely the network of genes implicated in stress is numerous and complex and that subsequent experiment will be required to validate project finding. The mapping of TCs provides meaningful knowledge regarding this network of genes. Functional annotation could be assigned to 1347 (out of 1906) TCs and 243 (out of 349) that colocalized within the 99% confidence interval of at least a single QTL. An abiotic stress response annotation could be assigned to 245 (out of 1347) and 85 (out of 243) TCs from outside (non-QTL) or within a QTL interval, respectively. Several key questions can be answered with this knowledge, including that putative stress-responsive genes will map within QTLs associated with stress-related phenotypic variation more frequently than with other genomic regions not associated with these QTLs. Mapping stress responsive QTLs in cotton [5–7] revealed a shared network of QTL intervals that control many different traits in response to stress. Because the QTL intervals examined represent a small fraction of the genome size, the number of TCs (or percentage of total) that mapped outside verse within a QTL was not expected to be equal. However, the percent that maps within (69.6%) or outside (70.9%) a QTL with putative function is important to show an equivalent representation of annotated genes in both classed regions (Table 2). The percent that maps within (35.0%) or outside (14.5%) a QTL with putative stress function is used to test our hypothesis (Table 2). If these numbers were equal or skewed (to intervals outside the QTLs) the research hypothesis would be rejected. However, the observations support the hypothesis and a simple binomial equation was used to calculate the probability of this degree of bias being observed if QTL and non-QTL regions are equally likely to contain stress genes. The very low likelihood (7.99 × 10^−15^) of this observation strongly suggests that the QTL intervals have a higher propensity to contain stress genes. So as QTL mapping alludes to common regions of the genome that explain the phenotypic variation to a variety of traits, those regions also appear to contain a higher number of putative stress genes. So these results show there is value to examine the structural position of putative stress genes with QTLs to study the network of stress genes.

Fifteen candidate genes (Levels I′ and II′) map to a single genomic region on Chr. 10 that contains QTLs for OP, CT, SC, and HI in response to drought stress (Tables 4(a) and 4(b)). This region is interesting because it harbors QTLs for physiological traits that influence productivity traits and contains candidate genes known to have a significant role in stress responses. In addition, this genomic region showed synteny with four* Arabidopsis *regions. Five* Arabidopsis* genes in these syntenic regions include genes that respond to salt stress, cold stress, and abscisic acid stimuli and genes that are involved in metabolic process of reactive oxygen species (Table 5). A strong relationship was found between QTLs for OP and those affecting SC, HI in multiple genomic regions [6]. Quantitative trait loci associated with lower OP and CT values were associated with increased productivity (SC and HI). These findings were further supported by significant phenotypic correlation. Osmotic adjustment has been shown to correlate with increased yield and dry matter production in various studies [22–25]. This suggests that OP plays a major role in influencing the productivity QTLs. The fifteen candidate genes associated with this region include genes that function in signal transduction (auxin-repressed protein, putative GTP-binding protein, and protein induced upon tuberization), transcription (transcription factor WRKY1, putative ethylene response factor, and homeobox-leucine zipper protein), and cell defense (putative thioredoxin and metallothionein-like protein) [26–32]. The gene expression analysis revealed that all candidate transcription factors were induced in cotton leaves under water-deficit stress. It is known that a complex network of transcription factors coordinates plant response to adverse environmental conditions [29]. The MYB proteins in* Arabidopsis *function as transcriptional activators in abscisic acid (ABA) inducible gene expression under water-deficit and salt stress [26].* Arabidopsis* MYB protein (At4g09460) has synteny with this QTL region and is homologous to the cotton candidate gene TC 7534. Most of the drought-inducible genes studied are induced by ABA [26]. The other candidate genes like thioredoxin and metallothionein-like protein play a central role in the regulation of reactive oxygen intermediates and abiotic stress signaling in* Arabidopsis* [29, 31]. This group of candidate genes may be involved in osmotic adjustment by osmotic-stress signaling leading to the expression of early response transcriptional activators, which then activate downstream stress tolerance effector genes [33] suggesting that the candidate genes associated with QTLs in this genomic region largely influence OP. Additionally,* Arabidopsis* genes which respond to osmotic stress, ABA, and other abiotic stresses showed association with the OP QTL on Chrs. 11 and 13.

Seven candidate genes (Levels I′ and II′) are associated with QTLs for SC, HI, and BW on Chr. 12. Included in this group are transcription factors associated with ABA-mediated stomatal movement and plant water balance (*AtGPA1*), heat shock (*Hsp20.1*), and transcriptional activation (zinc finger-like protein) [29, 34, 35]. The* Arabidopsis *syntenic regions contain three genes involved in signal transduction and in the metabolic process of oxygen and reactive oxygen. Moreover, these productivity QTLs on Chr. 05 also have syntenic regions with* Arabidopsis* which contain a considerable number of known stress responsive genes.

Five candidate genes (Levels I′ and II′) on Chr. 11 are associated with water use efficiency (*δ* ^13^C). Transcription initiation factor TFIID is involved in the regulation of gene expression and adaptation to osmotic stress [36]. Two proline-rich proteins, known to be important in reducing stress injury in plants [37], were associated with this QTL. Saranga et al. [6] found association between *δ* ^13^C and chlorophyll content (*Chl-a *and* Chl-b*) in two genomic regions (Chr. 22 and LGD05 of the tetraploid genome) that correspond to Chr. 8 on the Consensus Map. They found that QTL alleles associated with higher *δ* ^13^C under water-deficit conditions coincided with lower chlorophyll content. This QTL region is associated with ATP synthase, an enzyme that catalyzes the synthesis of ATP during photosynthesis and respiration [38]. In the chloroplast, ATP synthase utilizes the free energy released by electron transport and assumes an import role in regulating adjustment of the photosynthetic system to varying environmental conditions [38]. As chlorophyll content and *δ* ^13^C are associated with photosynthesis this candidate gene may have a role in influencing the QTLs for *δ* ^13^C and chlorophyll content in this genomic region.

Candidate genes for fiber quality QTLs (Levels I′, II and II′) on Chrs. 7 and 12 include epoxide hydrolase, a detoxification enzyme that removes reactive oxygen species during stress conditions [33], omega-3 fatty acid desaturase, a gene involved in cold stress tolerance [39], and a zinc finger-like protein, a gene suggested to play a role in reactive oxygen and abiotic stress signaling in* Arabidopsis* [29]. Signal transducer and transcription regulator genes in* Arabidopsis* have synteny with these QTLs. Two hypothetical proteins are also identified as candidate genes for these fiber traits, one induced in both root and leaf tissue and the other repressed in roots in our gene expression profiling. Since the microarray was developed mainly from fiber tissue these genes may be important in fiber development under water-deficit stress conditions.

Combining gene expression data, genetic mapping information, and physiological data is an important step towards understanding the genetics controlling the physiological responses that affect fiber production and quality under arid conditions. This strategy combines the use of a genome-wide approach to identify and isolate key candidate genes to specific regions of the genome, with the full benefits of a rich history of phenotypic data accumulated in several studies. In this study, candidate genes that may influence water stress-related QTLs in cotton have been identified using this strategy. Synteny between cotton and* Arabidopsis* made it possible to identify additional genes involved in stress response. These candidates, in addition to genes from other studies, represent putative functions that are critical during water-deficit stress response in cotton but warrant further functional testing to determine if they or related pathways are directly responsible or could be employed as targets for the improvement of agronomically desired traits for cotton production.

## Figures and Tables

**Table 1:** Cotton cDNA libraries used for EST assembly (details on these libraries can be accessed at http://www.agcol.arizona.edu/cgi-bin/pave/Cotton/index.cgi/).

Library source Tissue^a^	Library ID	Number of ESTs
Etiolated cotyledon	GH_ECOT, GH_ECT	5084
Seedling (control)	GH_SDL	1378
Seedling (drought stress)	GH_SDLD	589
Seedling (chilling stress)	GH_SDCH	207
Ovary (−3 to 1 dpa)	GH_FOX	6896
8–10 dpa boll (irrigated)	GH_MDI	946
8–10 dpa boll (drought stress)	GH_MDDS	988
15–20 dpa boll (irrigated)	GH_LDI	1428
15–20 dpa boll (drought stress)	GH_LDDS	1014
Mature stem	GH_STEM	6588
Total		25,118

^a^Seedling libraries include cDNAs from root and shoot tissues.

dpa is the days after anthesis.

**Table 2:** Association of Mapped TCs with QTLs.

Cotton QTL^a^	QTL position^b^		TCs mapped		Differentially expressed genes
	Chr.	Confidence interval (cM)	Total	Putative stress function	
OP	1	86.6	13	0	—
*δ* ^13^C, FF	2	64–77.6	31	1	1
*Chl-a*, *δ* ^13^C	3	76.0–76.6	6	5	1
OP, *Chl-a *	4	29–93.5	27	4	3
BW, DM, SC, HI	5	36.7–106.2	23	2	3
BW, HI	6	29.7–59.7	7	0	1
FS	6	138.8–139.4	2	0	—
FS	7	91.2–131.4	12	1	2
SC, HI, BW, OP, *δ* ^13^C	7	167–217	50	20	5
*Chl-a*, *Chl-b*, *δ* ^13^C	8	44.9–52.3	10	2	1
OP, SC, HI, CT	10	77.5–122.2	57	20	8
*δ* ^13^C	11	44.0–56.7	17	6	5
OP	11	94.6–104.9	14	0	—
SC, HI, BW	12	0.0–25.5	16	5	1
FL	12	38.8–43.1	32	8	2
OP, SC, HI, *Chl-a *	13	42.6–50.7	26	5	1
OP, BW	13	131.5–149.7	6	6	—

^a^Physiology traits studied include OP (osmotic potential), *δ* ^13^C (carbon isotope ratio), *Chl* (chlorophyll content), and CT (canopy temperature). Production traits studied were BW (boll weight), SC (seed cotton yield), HI (harvest index), and DM (dry matter weight). Fiber quality traits comprise FF (fiber fineness), FS (fiber strength), and FL (fiber length).

^b^Rong et al., 2007 [15].

**Table 3:** Association of stress-related genes with stress-related QTL.

Mapped TC	Total	Percentage putative function^a^	Percentage putative stress function^b^
Outside QTL regions	1557	70.9% (1104)	14.5% (160)
Within QTL regions	349	69.6% (243)	35.0% (85)
Total	1906	70.7% (1347)	18.2% (245)

^a^Percentage of the total TCs with putative function.

^b^Percentage of the TCs with putative function that have stress-related function.

**Table:** (a) TCs with stress related gene ontology and mapped to stress QTL

ID^a^	STS marker	Chr.	Position	QTL	Expression^b^	Putative function	Candidate category^c^
TC_12018	Coau2L06	2	75.8	*δ* ^13^C, FF		Calcium-dependent protein kinase 2	I′
TC_1043, 2901, 4773, 4777, 8848	pAR04H03	3	76.6	*Chl-a*, *δ* ^13^C		Xyloglucan endotransglucosylase/hydrolase protein	I′
TC_1191, 2292, 3741, 4297	pAR0594	4	87.2	*Chl-a *	↑ leaf	Putative transport protein subunit	II′
TC_15220, 5078	P05-06	5	36.7	DM	↑ leaf	Probable WRKY transcription factor	II′
TC_3299	pAR0207b	7	106.4	FS	↑ root	Putative linker histone H1 variant protein	II′
TC_3097, 6916, 8776	pAR03D05	7	172.1	SC		Putative CCR4 transcription complex	I′
TC_11788, 6599, 6600	Gafb22M15c	7	187.9	HI, BW, SC		Probable xyloglucan endotransglucosylase	I′
TC_12140, 370	pAR0922	7	202.3	HI, OP		stress-induced protein	I′
TC_11870, 12131, 4218, 5814, 9408	Unig24G08d	7	205.6	HI, OP		Luminal binding protein	I′
TC_3660, 5958	pGH843	7	207.4	HI, OP, *δ* ^13^C	↓ leaf	Ubiquitin/ribosomal protein	II′
TC_2075, 2076, 2077, 8440, 9924	pAR3-26	7	210.3	HI, OP, *δ* ^13^C	↓ leaf	Zinc finger	II′
TC_15720, 6936	Unig28D04	8	45.0	*Chl-a*, *Chl-b*, *δ* ^13^C	↓ leaf	ATP synthase	II′
TC_241, 8023, 8765	Gate4CE02	10	95.6	OP, SC, HI, CT	↑ leaf, ↓ root	Transcription factor WRKY1	II′
TC_1092	pAR0836	10	95.6	OP, SC, HI, CT		Photosystem II oxygen-evolving complex	I′
TC_4882	Gate4AH05	10	97.4	OP, SC, HI, CT	↑ root	Putative thioredoxin m2	II′
TC_7520, 7521, 7522, 7523, 7524	pAR0783	10	100.6	OP, SC, HI, CT		Auxin-repressed 12.5 KD protein	I′
TC_2988, 4865, 5435, 7627	Coau4J19	10	106.7	OP, SC, HI, CT		Protein induced upon tuberization	I′
TC_13398	Unig26D12	10	106.7	OP, SC, HI, CT		Putative GTP-binding protein	I′
TC_1142	pAR0949	10	112.7	OP, SC, HI, CT		Metallothionein-like protein	I′
TC_9516	pGH663	10	112.9	HI	↑ leaf	Homeobox-leucine zipper protein	II′
TC_159, 6239	pAR0211	10	114.5	HI		Heat shock protein 70	I′
TC_7534	Gate4CE05a	10	122	HI		MYB-like DNA-binding domain protein	I′
TC_3646, 3647, 3648	Gate1CE04b	11	45.7	*δ* ^13^C	↑ leaf	Ubiquitin extension protein	II′
TC_10672, 5735	pAR08A01	11	45.7	*δ* ^13^C	↑ root	Putative proline-rich protein	II′
TC_14408	Unig26E05	11	52.9	*δ* ^13^C	↑ leaf	Transcription initiation factor TFIID subunit 9	II′
TC_4402, 5648, 7125, 7127, 7134	Gate1CD11b	12	0.0	SC, HI, BW		Transducer (GPA1), *Arabidopsis thaliana* GPA1	I′
TC_12229, 12900, 12901	Gate4CG05a	12	38.8	FL		Soluble epoxide hydrolase	I′
TC_3419	Gate4CA09b	12	39.6	FL	↓ leaf	Zinc finger-like protein	II′
TC_11086	Gate4AE08b	12	40.4	FL		Hsp20.1 protein	I′
TC_3376, 7396, 7397	pVNC146b	12	41.5	FL		Omega-3 fatty acid desaturase, chloroplast precursor	I′
TC_3270, 3271, 3272, 4970, 8481	Gate4CG12	13	50.7	SC, HI		Thioredoxin-like protein 1	I′
TC_15659	Coau2L21	13	131.5	OP, BW		Adenylyl cyclase associated protein	I′
TC_4763, 8193, 8194, 8196, 8198	Gate1AA03	13	135.0	OP, BW		Glycine-rich RNA-binding protein	I′

**Table:** (b) Differentially expressed genes mapped to stress QTL

ID^a^	STS Marker	Chr	Position	QTL	Expression^b^	Putative Function	Candidate Category^c^
Cotton12_00001_132	Gate3CC07a	2	76.2	*δ* ^13^C, FF	↓ leaf	Putative epimerase/dehydratase	II
Cotton12_00007_02	Coau1O15	3	76.0	*Chl-a *	↑ root, ↑ leaf	Unknown protein	II
Cotton12_18270_01	Gate4BG06a	4	74.4	*Chl-a *	↓ leaf	Homeobox-leucine zipper protein	II′
Cotton12_00128_02	Gate2BC04	4	103.5	*Chl-a *	↓ leaf	Unknown protein	II
Cotton12_14391_01	P05-06	5	36.7	DM	↓ leaf	F23H11.2 protein (At1g59710/T30E16_31)	II
Cotton12_07910_01	W07a	5	105.3	SC, HI, BW, FF	↓ leaf	Gossypium putative major latex-like protein	II
Cotton12_25608_01	A1737	6	49.8	HI, BW	↓ root	Hypothetical protein	II
Cotton12_00659_01	pAR0537	7	97.0	FS	↓ leaf	Putative glycine hydroxymethyltransferase	II
Cotton12_16738_01	pGH505	7	131.4	FS	↓ leaf	Hypothetical protein	II
Cotton12_00017_01	P01-42	7	192.6	SC, HI, BW	↓ leaf	RNA-binding protein	II
Cotton12_12667_01	P02-45	7	210.1	HI, OP, *δ* ^13^C	↑ leaf	Expressed protein	II
Cotton12_04174_01	Unig06B11	10	96.5	OP, SC, HI, CT, FS	↑ leaf	Hypothetical protein	II
Cotton12_04395_01	pAR10F02	10	100.6	OP, SC, HI, CT, FS	↑ leaf	Putative ethylene response factor ERF3a	II′
Cotton12_00370_02	pAR0602	10	108.2	OP, SC, HI, CT, FS	↓ leaf	ATP synthase gamma chain, chloroplast precursor	II′
Cotton12_01999_01	Gate1DD01	10	112.7	OP, SC, HI, CT, FS	↓ leaf	Hypothetical protein	II
Cotton12_35202_01	G1099	10	116.2	OP, SC, HI, CT, FS	↓ leaf	Glutamyl tRNA amidotransferase, subunit A	II
Cotton12_02930_01	Gafb28K14	11	45.7	*δ* ^13^C	↑ leaf	4-Coumarate:CoA ligase	II′
Cotton12_06698_01	pAR01D04	11	54.4	*δ* ^13^C	↑ root	Proline-rich protein APG-like	II′
Cotton12_18985_01	Gate4BD10a	12	22.3	SC, HI, BW	↓ root, ↑ leaf	Hypothetical protein	II
Cotton12_18065_01	Unig22D08	12	38.8	FL	↓ root	Hypothetical protein	II
Cotton12_19658_01	W11	13	47.6	OP, SC, HI, *Chl-a *	↑ root	Hypothetical protein	II

**Table:** (c) GeneS unique to stress library and are differentially expressed

ID^a^	STS Marker	Chr	Position	QTL	Expression^b^	Putative Function	Candidate Category^c^
Cotton12_27963_01					↑ leaf	Fiber protein Fb17 (Fragment)	II
Cotton12_23787_01					↑ leaf	Hypothetical protein At2g04690	II
Cotton12_07697_01					↓ leaf	Hypothetical protein At5g65650	II
Cotton12_26110_01					↓ leaf	Hypothetical protein F3L17.50	II
Cotton12_31394_01					↑ root	Hypothetical protein OJ1520_C09.39	II
Cotton12_34263_01					↑ leaf	Inner membrane metabolite transport protein	II
Cotton12_35169_01					↑ leaf	NAC-domain protein	II′
Cotton12_22936_01					↓ leaf	Putative flavonol 3-O-glucosyltransferase	II′
Cotton12_05870_01					↑ leaf	OSJNBa0086B14.2 protein	II
Cotton12_32869_01					↑ root	Q9XEU0_DATGL	II
Cotton12_30514_01					↑ leaf	Q9XI23_ARATH	II
Cotton12_38044_01					↑ leaf	Syringolide-induced protein 1-3-1B	II

**Table:** (d) Gene unique to stress library and mapped to a stress QTL

ID^a^	STS Marker	Chr	Position	QTL	Expression^b^	Putative Function	Candidate Category^c^
Cotton12_18881_01	CMS14	1	88.6	OP		No Hit	II

^a^Candidate genes are listed by TC or EST ID. Homologous STS Marker have been provided.

^b^Stress induced (↑) and stress repressed (↓) expression in response to drought conditions has been provided.

^c^LevelS I and II candidate genes are indicated. A prime (′) designation on each category indicates stress-related gene ontology.

**Table 5:** Cotton QTLs with *Arabidopsis* syntenic regions and associated orthologous stress related genes.

Cotton QTL region^a^	*Arabidopsis* syntenic segments^b^	*Arabidopsis* genes	Gene function
*δ* ^13^C, FF (Chr. 2; 64–77.6 cM)	D05.99	AT1G23130	Defense response

OP, *Ch-a* (Chr. 4; 29–93.5 cM)	D03.92	AT1G09250	Regulation of transcription
	D06.26	AT1G31930	G-protein coupled receptor protein signaling pathway
	D08.54	AT3G21240	Response to UV, response to wounding
	D11.56	AT2G45070	Protein transporter activity

BW, DM, SC, HI (Chr. 5; 36.7–106.2 cM)	D05.114	AT1G77760	Response to light stimulus
	D15.111	AT3G16940	Calmodulin binding, transcription regulator activity
	D23.119	AT5G09800	Protein ubiquitination
	D01.119	AT4G00720	Kinase activity
	D01.121	AT1G23740	Zinc ion binding
	D02.120	AT2G32250	Response to red or far red light, zinc ion binding
	D10.124	AT2G17290	Abscisic acid mediated signaling, regulation of stomatal movement
	D11.122	AT2G35890	Calmodulin-dependent protein kinase activity
	D12.116	AT5G28000	Response to biotic stimulus, defense response
	D12.121	AT3G05530	Calmodulin binding, ubiquitin-dependent protein catabolic process
	DS07.115	AT5G23420	Regulation of transcription
	DS07.115	AT5G23540	Ubiquitin-dependent protein catabolic process

FS (Chr. 7; 91.2–131.4 cM)	D01.150	AT1G03010	Response to light stimulus, signal transducer activity
	D11.66	AT2G37250	Nucleotide kinase activity
	D11.66	AT2G37710	Kinase activity
	D12.153	AT3G02940	Response to salicylic acid stimulus, regulation of transcription
	D23.149	AT5G64330	Signal transducer activity
	D23.33	AT5G65020	Calcium-dependent phospholipid binding

SC, HI, BW, OP (Chr. 7; 167–217 cM)	D02.67	AT2G31750	Abscisic acid glucosyltransferase activity
	D03.143	AT1G12270	Response to stress
	D09.70	AT5G41360	Response to UV-B
	D09.70	AT5G42020	Response to heat
	D15.164	AT4G14130	Xyloglucan endotransglycosylase-related protein
	D15.164	AT3G23830	Response to osmotic stress, response to salt stress
	DS02.34	AT3G26700	Kinase activity
	DS02.69	AT3G26700	Kinase activity

*Ch-a, Chl-b*, *δ* ^13^C (Chr. 8; 44.9–52.3 cM)	D06.183	AT1G31930	G-protein coupled receptor protein signaling pathway
	D06.29	AT1G32640	Response to desiccation, response to abscisic acid stimulus
	D10.73	AT2G22800	Regulation of transcription, DNA-dependent
	D21.17	AT5G47030	ATP synthesis coupled proton transport

OP, SC, CT, HI (Chr. 10; 77.5–122.2 cM)	D10.232	AT4G37930	Oxygen and reactive oxygen species metabolic process
	D10.232	AT5G20720	Response to cold, calmodulin binding
	D17.231	AT2G43220	Zinc ion binding
	D07.243	AT4G09460 (MYB2.1)	Response to abscisic acid stimulus, response to salt stress, response to ethylene stimulus
	D11.165	AT3G61150	Regulation of transcription, DNA-dependent

BW, HI, SC (Chr. 12; 0–25.5 cM)	D10.175	AT4G37930	Oxygen and reactive oxygen species metabolic process
	D17.269	AT3G43810	Calcium-mediated signaling

HI, SC (Chr. 13; 42.6–50.7 cM)	D03.88	AT1G08810	Stomatal movement, response to water deprivation, abscisic acid stimulus, salt stress, salicylic acid stimulus
	D12.292	AT5G18100	Oxygen and reactive oxygen species metabolic process copper, zinc superoxide dismutase activity

OP (Chr. 13; 131.5–149.7 cM)	D10.303	AT4G37930	Oxygen and reactive oxygen species metabolic process
	D10.303	AT4G38230	Calmodulin-dependent protein kinase activity
	D10.304	AT2G21510	Heat shock protein binding
	D11.187	AT2G37290	Regulation of Rab GTPase activity

^a^Chromosome and location of QTLs are shown in brackets.

^b^ *Arabidopsis α* duplicates [16].
